# Supplementary material for: Characteristics of Transposable Element Exonization within Human and Mouse
Source: PLoS One. 2010 Jun 1;5(6):e10907. doi: 10.1371/journal.pone.0010907 (PMC2879366; doi:10.1371/journal.pone.0010907)
Supplement: Table S1 — SNPs in non-canonical splice sites of exonized transposed elements in the human genome as well as in the mouse genome resulting in a canonical splice site. Given are the gene id, the chromosome and strand on which the SNP is located, the start and end of the exon which derived from the transposed element, the transposed element's family, the SNP id and the alleles of the SNP and the position at which the SNP is located (always seen from the exon, that is, 1st position of acceptor indicates the base which is located nearest to the splice site). (0.05 MB DOC) [file pone.0010907.s001.doc]

Table S1:

| gene id | chr./strand | start–end | TE | SNP info | position | Chimp/Rhesus |
| --- | --- | --- | --- | --- | --- | --- |
| human |  |  |  |  |  |  |
| BI561944 | chr22/+ | 46079860–978 | Alu | rs5767715 (C/G) | 1st pos. don. | Chimp – G  Rhesus- G |
| BX114231 | chr6/+ | 55453454–517 | L1 | rs13193836 (A/G) | 1st pos. don. | Chimp – A  Rhesus- A |
| DA324777 | chr17/- | 39316977–7049 | L1 | rs231518 (A/G) | 1st pos. don. | Chimp – G  Rhesus - A |
| AA431656 | chr8/+ | 13240453–633 | L2 | rs1729111 (G/T) | 1st pos. acc. | Chimp – T  Rhesus - T |
| AA451797 | chr10/+ | 109138035–158 | L2 | rs2297856 (A/T) | 2nd pos. don. | Chimp – A  Rhesus –A  Mouse –A  Rat -A |
| BX101391 | chr8/+ | 20857475–625 | LTR | rs491797 (A/G) | 1st pos. don. | Chimp – G  Rhesus – G  Mouse - T |
| BF922696 | chr1/- | 158300819–989 | LTR | rs430178 (C/G) | 1st pos. don. | Chimp – G  Rhesus - G |
| AA332456 | chr15/- | 18899282–340 | DNA | rs466250 (A/G) | 1st pos. don. | Chimp – A  Rhesus – A  Mouse - A |
| AL037953 | chr15/+ | 26704727–785 | DNA | rs466250 (A/G) | 1st pos. don. | Chimp –A  Rhesus –A  Mouse - A |
| BX114101 | chr15/- | 48954060–215 | DNA | rs1810098 (G/T) | 2nd pos. don. | Chimp – T  Rhesus - T |
| BU567215 | chr9/- | 49140–230 | MIR | rs2121442 (G/T) | 1st pos. don. | Chimp – T  Rhesus – T  Mouse – T |
| BU567215 | chr9/- | 66944051–141 | MIR | rs2121442 (G/T) | 1st pos. don. | Chimp – T  Rhesus – T  Mouse – T |
