# Supplementary material for: Characteristics of Transposable Element Exonization within Human and Mouse
Source: PLoS One. 2010 Jun 1;5(6):e10907. doi: 10.1371/journal.pone.0010907 (PMC2879366; doi:10.1371/journal.pone.0010907)
Supplement: Table S2 — Population frequency data for the SNPs which changed a non-canonical splice site into a canonical one while the other splice site was already canonical. Given is the SNP id along with the alleles and the position where this SNP occurred as well as the frequency data. Here, the homozygosity for the first allele, the heterozygosity, the homozygosity for the second allele, the Hardy-Weinberg proportions as well as the frequencies for each of the alleles are given. CEPH-European, HISP-Hispanic, AD-African American, CEU-European, HCB-Asian, JPT-Asian, YRI-Sub-Saharan African, HWP-Hardy-Weinberg proportions. (0.10 MB DOC) [file pone.0010907.s002.doc]

Table S2:

| Genotype detail | Alleles |  |  |  |  |  |  |
| --- | --- | --- | --- | --- | --- | --- | --- |
| SNP id | population | A/A | A/G | G/G | HWP | A | G |
| rs231518 (A/G, donor 1st position) | CEU | 0.033 | 0.35 | 0.617 | 0.655 | 0.208 | 0.792 |
|  | HCB |  |  | 1 |  |  | 1 |
|  | JPT |  |  | 1 |  |  | 1 |
|  | YRI |  | 0.067 | 0.933 | 1 | 0.033 | 0.967 |
| rs390369 (A/G, acceptor 1st position) | CEU | 0.102 | 0.39 | 0.508 | 0.655 | 0.297 | 0.703 |
|  | HCB | 0.422 | 0.489 | 0.089 | 0.527 | 0.667 | 0.333 |
|  | JPT | 0.444 | 0.422 | 0.133 |  | 0.656 | 0.344 |
|  | YRI | 0.183 | 0.317 | 0.5 | 0.025 | 0.342 | 0.658 |
| rs2385796 (A/G, donor 1st position) | CEU | 0.2 | 0.533 | 0.267 | 0.584 | 0.467 | 0.533 |
|  | HCB | 0.467 | 0.422 | 0.111 | 1 | 0.678 | 0.322 |
|  | JPT | 0.591 | 0.341 | 0.068 | 0.752 | 0.761 | 0.239 |
|  | CEU | 0.083 | 0.467 | 0.45 | 0.584 | 0.317 | 0.683 |
| rs6586266 (A/G, acceptor 2nd position) | HCB | 0.511 | 0.333 | 0.156 | 0.15 | 0.678 | 0.322 |
|  | JPT | 0.432 | 0.477 | 0.091 | 0.655 | 0.67 | 0.33 |
|  | YRI |  | 0.085 | 0.915 | 0.752 | 0.042 | 0.958 |
|  |  | G/G | G/T | T/T | HWP | G | T |
| rs17843996 (G/T, donor 1st position) | AFRICAN | 0.857 | 0.143 |  | 0.752 | 0.929 | 0.071 |
|  | EUROPEAN | 0.476 | 0.429 | 0.095 | 1 | 0.69 | 0.31 |
|  |  |  |  |  |  |  |  |
| rs1366046 (G/T, donor 1st position) | CEU | 0.119 | 0.576 | 0.305 | 0.15 | 0.407 | 0.593 |
|  | HCB | 0.455 | 0.432 | 0.114 | 1 | 0.67 | 0.33 |
|  | JPT | 0.467 | 0.422 | 0.111 |  | 0.678 | 0.322 |
|  | YRI | 0.051 | 0.237 | 0.712 | 0.251 | 0.169 | 0.831 |
| rs1810098 (G/T, donor 2nd position) | CEPH |  |  |  |  | 0.02 | 0.98 |
|  |  |  |  |  |  |  |  |
|  |  |  |  |  |  |  |  |
|  |  | C/C | C/G | G/G | HWP | C | G |
| rs1047206 (C/G, donor 1st position) | HISP | 0.667 | 0.267 | 0.067 |  | 0.8 | 0.2 |
|  | CEPH | 0.706 | 0.294 |  |  | 0.853 | 0.147 |
|  | AD | 0.917 | 0.083 |  |  | 0.958 | 0.042 |
|  | ASIAN | 0.864 | 0.136 |  |  | 0.932 | 0.068 |
| rs949250 (C/G, donor 1st position) | YRI | 0.224 | 0.483 | 0.293 | 1 | 0.466 | 0.534 |
|  | AFRICAN | 0.118 | 0.353 | 0.529 | 0.584 | 0.294 | 0.706 |
|  |  |  |  |  |  |  |  |
| rs430178 (C/G, donor 1st position) | CEPH |  |  |  |  | 0.88 | 0.12 |
|  |  |  |  |  |  |  |  |
|  |  |  |  |  |  |  |  |
|  |  | A/A | A/T | T/T | HWP | A | T |
| rs2297856 (A/T, donor 2nd position) | CEU | 0.351 | 0.561 | 0.088 | 0.15 | 0.632 | 0.368 |
|  | HCB | 0.022 | 0.244 | 0.733 | 1 | 0.144 | 0.856 |
|  | JPT | 0.044 | 0.111 | 0.844 | 0.02 | 0.1 | 0.9 |
|  | YRI | 0.172 | 0.483 | 0.345 | 1 | 0.414 | 0.586 |
|  | Caucasian |  |  |  |  | 0.67 | 0.33 |
|  | Chinese |  |  |  |  | 0.2 | 0.8 |
|  | Japanese |  |  |  |  | 0.09 | 0.91 |
